# Polyethylene Glycol Preconditioning: An Effective Strategy to Prevent Liver Ischemia Reperfusion Injury

**DOI:** 10.1155/2016/9096549

**Published:** 2016-02-15

**Authors:** Mohamed Bejaoui, Eirini Pantazi, Maria Calvo, Emma Folch-Puy, Anna Serafín, Gianfranco Pasut, Arnau Panisello, René Adam, Joan Roselló-Catafau

**Affiliations:** ^1^Experimental Pathology Department, Institute of Biomedical Research of Barcelona (IIBB), CSIC, Rossello 161, Barcelona, 08036 Catalonia, Spain; ^2^Advanced Optical Microscopy Unit CCiTUB, Science and Technology Center, Faculty of Medicine, University of Barcelona, C/Casanova 143, Barcelona, 08036 Catalonia, Spain; ^3^Platform of Laboratory Animal Applied Research, Barcelona Science Park, Barcelona, 08028 Catalonia, Spain; ^4^Pharmaceutical and Pharmacological Sciences Department, University of Padova, 35131 Padova, Italy; ^5^Veneto Institute of Oncology (IOV), IRCCS, 35128 Padova, Italy; ^6^Hepato-Biliary Centre, Paul Brousse Hospital, Inserm U776, Paris-Sud University, Villejuif, 75008 Paris, France

## Abstract

Hepatic ischemia reperfusion injury (IRI) is an inevitable clinical problem for liver surgery. Polyethylene glycols (PEGs) are water soluble nontoxic polymers that have proven their effectiveness in various* in vivo* and* in vitro* models of tissue injury. The present study aims to investigate whether the intravenous administration of a high molecular weight PEG of 35 kDa (PEG 35) could be an effective strategy for rat liver preconditioning against IRI. PEG 35 was intravenously administered at 2 and 10 mg/kg to male Sprague Dawley rats. Then, rats were subjected to one hour of partial ischemia (70%) followed by two hours of reperfusion. The results demonstrated that PEG 35 injected intravenously at 10 mg/kg protected efficiently rat liver against the deleterious effects of IRI. This was evidenced by the significant decrease in transaminases levels and the better preservation of mitochondrial membrane polarization. Also, PEG 35 preserved hepatocyte morphology as reflected by an increased F-actin/G-actin ratio and confocal microscopy findings. In addition, PEG 35 protective mechanisms were correlated with the activation of the prosurvival kinase Akt and the cytoprotective factor AMPK and the inhibition of apoptosis. Thus, PEG may become a suitable agent to attempt pharmacological preconditioning against hepatic IRI.

## 1. Introduction

Ischemia reperfusion injury (IRI) is inherent to surgical procedures such as liver resection and liver transplantation. The deleterious effects caused by IRI are the main cause of graft primary nonfunction and dysfunction [1]. Many strategies have been developed to protect against IRI such as ischemic preconditioning (IPC) and the use of different drugs. However, these strategies did not prove their effectiveness in clinical setting and efficient treatments are still lacking.

Polyethylene glycols (PEGs) are water soluble nontoxic polymers with different molecular weights and properties that have been extensively used in numerous applications (cosmetic, foods, pharmacy, and biomedicine) [2]. Also, PEGs have been found to exert beneficial effects in various* in vivo* and* in vitro* models of tissue injury [3–8]. Recently, it has been demonstrated that intravenous administration of high molecular weight PEG of 20 and 35 kDa protected rat heart against reperfusion injury and steatotic livers against cold IRI, respectively [9, 10]. The protective effects were associated with decreased vascular permeability, decreased oxidative stress, and inhibition of cell death [8, 11].

The aim of the present study is to examine the potential benefits of prophylactic intravenous administration of PEG 35 in order to prevent warm IRI in rat liver as well as to investigate the underlying mechanisms.

## 2. Materials and Methods

### 2.1. Animals

Male Sprague Dawley rats (250–300 g) were purchased from Charles River (France) and housed in a temperature and humidity controlled room under a constant 12-hour light/dark cycle. Animals had free access to water ad libitum and rat chow (standard laboratory pelleted formula A04, Panlab, Barcelona, Spain). This study was performed in accordance with European Union Directive 2010/63/EU for animal experiments and approved by the Ethics Committees for Animal Experimentation of the University of Barcelona (number 696/14).

### 2.2. Surgical Procedure

All the procedure was performed under isoflurane inhalation (induction dose of 5% and maintenance dose of 1.5–2%). Also, analgesia was applied before surgery by subcutaneous injection of buprenorphine at the dose of 0.05 mg/kg. After laparotomy, ischemia was induced by occlusion of the hepatic artery and portal vein of the left and median lobes using an atraumatic microvascular clip (70% ischemia). After one hour of ischemia, liver reperfusion was established by removal of the clamp and the abdomen was sutured. Then, rats were kept in clean cages with free access to water and standard rodent chow. After 2 h of reperfusion, animals were sacrificed by cervical dislocation under isoflurane anaesthesia for blood and tissue collection. Sham operated rats underwent the same procedure without vascular clamping.

### 2.3. Drug Treatment

PEG 35 was kindly provided by Institute Georges Lopez (IGL). PEG 35 was dissolved in phosphate buffer saline (PBS) and administrated 10 min before liver ischemia by intravenous bolus via the penile vein at the concentration of 2 mg/kg or 10 mg/kg using PEG 35 solution of 1 g/L and 5 g/L, respectively. For intravital microscopy study, PEG 35 was fused with fluorescein (PEG-FITC) as previously described by Mero et al. [12].

### 2.4. Experimental Groups

Rats were randomly distributed into four groups as follows.

*Group 1 (Control: n = 6)*. Midline incision was performed and hepatic pedicle was dissected. Then, 500 *μ*L of PBS was injected intravenously via the penile vein and abdomen was sutured. After 2 h, rats were sacrificed for blood and sample collection.

*Group 2 (IR 2 h, n = 6)*. Rats were pretreated with 500 *μ*L of PBS intravenously and then subjected to one hour of ischemia followed by 2 h of reperfusion. Then, animals were sacrificed and plasma and liver samples were collected.

*Group 3 (PEG 2 mg/kg, n = 6)*. It is the same as group 2 but rats were pretreated with intravenous administration of PEG 35 at the dose of 2 mg per kg body weight.

*Group 4 (PEG 10 mg/kg, n = 6)*. It is the same as group 2 but rats were pretreated with intravenous administration of PEG 35 at the dose of 10 mg per kg body.

### 2.5. Biochemical Determinations

#### 2.5.1. Hepatic Injury

Plasma levels of alanine aminotransferase (ALT) and aspartate aminotransferase (AST) were measured using a commercial kit from RAL (Barcelona, Spain) according to manufacturer's protocol.

#### 2.5.2. Glutamate Dehydrogenase Activity

GLDH activity was determined using a commercial kit (GLDH, Randox laboratories Ltd., Crumlin, UK) by quantifying the decrease in absorbance at 340 nm according to the manufacturer's protocol.

#### 2.5.3. Determination of Nitrites and Nitrates

Nitric oxide levels were measured as nitrate plus nitrite (NOx) in tissue samples using a commercial colorimetric assay kit (Cayman Chemical Co., Ann Arbor, MI, USA).

### 2.6. Western Blot Analysis

Liver tissue was homogenized in HEPES buffer and 50 *μ*g of protein was separated on 6–10% SDS-PAGE gels and transferred to PVDF membranes. Membranes were then incubated overnight at 4°C using the following antibodies: anti-eNOS (BD Transduction Laboratories, Lexington, KY, USA), anti-phosphorylated Akt, anti-total and anti-phosphorylated AMPK (Cell Signaling Technology Inc., Beverly, MA, USA), and anti-*β*-actin (Sigma Chemical, St. Louis, MO, USA). The corresponding secondary antibody was then added for 1 hour at room temperature and membranes were developed using the enhanced chemiluminescence reagents from Avision (Advansta, Menlo Park, CA, USA). Signals were quantified by scanning densitometry using the Quantity One software for images analysis. Results were expressed as densitometric ratio between the protein of interest and the correspondent Control (*β*-actin, total AMPK, and total Akt).

### 2.7. Histology

Formalin-fixed paraffin-embedded liver tissues were cut in 5 *μ*m sections and stained with hematoxylin and eosin according to standard procedures. Images were analysed by an independent investigator in blind manner.

### 2.8. F-Actin/G-Actin Ratio Measurements

To analyse the levels of F-actin and G-actin, liver samples were homogenized with PHEM buffer (60 mM Pipes, 20 mM HEPES, 10 mM EGTA, 2 mM MgCl_2_, 1% Triton X-100, and pH 7.0) and ultracentrifuged (48.000 rcf) at 4°C for 5 min to separate both fractions. Supernatant, containing G-actin, was collected; the F-actin pellet was washed twice with cold PHEM buffer and then dissolved in 1x SDS sample buffer. Equivalent amounts of proteins were separated by 10% SDS-PAGE, and F-actin and G-actin were determined by western blot and quantified by scanning densitometry. The F-actin/G-actin ratio was calculated.

### 2.9. Intravital Microscopy

Rats were anesthetized with isoflurane inhalation, laparotomized, and put in a prone position over a cover slip mounted on the stage of Leica TCS SP5 resonant scan multiphoton confocal microscope (Leica Microsystems Heidelberg GmbH) equipped with an incubation system with temperature control, HCX IR APO L 25x water immersion objective (Numerical Aperture 0.95), resonant scanner at 8000 lines/s, and a near-infrared Titanium:Sapphire laser (MaiTai, Spectra-Physics) for two-photon excitation running at 800 nm.

The following vital dyes were injected intravenously as indicated: Hoechst 33342 trihydrochloride (12 mg/kg, Invitrogen, H3570) for DNA-nuclei staining; Rhodamine 123 (0.11 mg/kg, Sigma, R8004) for mitochondrial membrane potential dye; Evans Blue (20 mg/kg, Sigma, E2129-10), a bulk fluid-phase albumin marker that enhances contrast of plasma; and PEG 35 conjugated with fluorescein isothiocyanate (PEG-FITC).

Images were acquired with resonant scan at 8000 lines/second.

### 2.10. Confocal Fluorescence Microscopy

Liver was fixed, cryoprotected with sucrose, embedded in OCT, and frozen on a copper plate on dry ice. Ten-micrometer cryosections were cut in a cryostat and postfixed in 4% buffered paraformaldehyde for 10 min and then permeabilized with PBS containing 0.1% Triton X-100 and 1% BSA for 30 min. For actin visualization, the slides were incubated with TRITC-Phalloidin (dilution 2 *μ*M, Sigma) in PBS with 1% BSA and 0.2% Triton X-100 for 30 min. Slides were washed three times for 15 min with PBS. The last PBS wash included Hoechst 33342 (dilution 1 mM, Invitrogen). Finally, cryosections were mounted using Mowiol (Calbiochem). Confocal images were acquired with Leica TCS SP5 laser scanning microscope. Hoechst 33342 and Phalloidin-A555 images were acquired sequentially using 405 and 561 nm laser lines. The confocal pinhole was set at 1 airy unit and when 3D reconstruction was required stacks of images every 0.3 mm were acquired. The hepatocytes size (in *μ*m^2^) and circularity as (4*π* × Area)/Perimeter^2^ (based on Phalloidin staining) were quantified on ImageJ. The red channel (Phalloidin-A555 staining) was processed to segment hepatocytes. Hepatocytes were selected and size and circularity were measured (in 1.5 mm^2^ of each sample). A value of 1.0 indicated a perfect circle; as the value approached 0.0, it indicated a more polyhedral shape.

### 2.11. Statistical Analysis

Data are expressed as means ± standard error and were compared statistically by the one-way analysis of variance, followed by the Tukey test (GraphPad Prism software). *P* < 0.05 was considered significant.

## 3. Results

**Figure 1:**
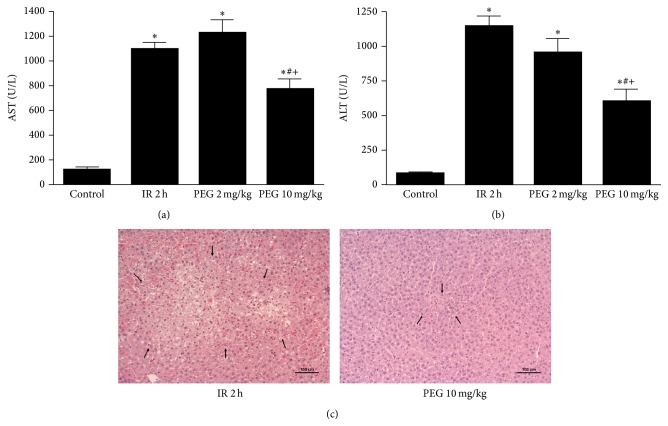
Hepatic injury after ischemia reperfusion. PEG 35 at 10 mg/kg decreases AST (a) and ALT (b) levels and the number of necrotic areas as shown by histological findings (eosin/hematoxylin staining) (c). Data represent mean ± SEM. ^*∗*^ *P* < 0.05 versus Control, ^#^ *P* < 0.05 versus IR 2 h, and ^+^ *P* < 0.05 versus PEG 2 mg/kg.

In order to evaluate the effect of PEG 35 in liver IRI, we firstly determined the liver damage through transaminases levels and hepatic histology after 1 hour of ischemia followed by 2 hours of reperfusion. As shown in Figures 1(a) and 1(b), IR group led to significant increases in transaminases levels compared to Control group, which was prevented when rats were pretreated with intravenous injection of PEG 35 at 10 mg/kg. By contrast, no significant differences were observed when PEG 35 at 2 mg/kg was administered. Histological findings shown in Figure 1(c) were in accordance with liver injury parameters. Animals subjected to IR showed extensive areas of coagulative hepatic necrosis with disruption of hepatic cords and haemorrhage randomly distributed throughout the hepatic parenchyma. PEG at 10 mg/kg reduced the extent and the number of necrotic areas.

**Figure 2:**
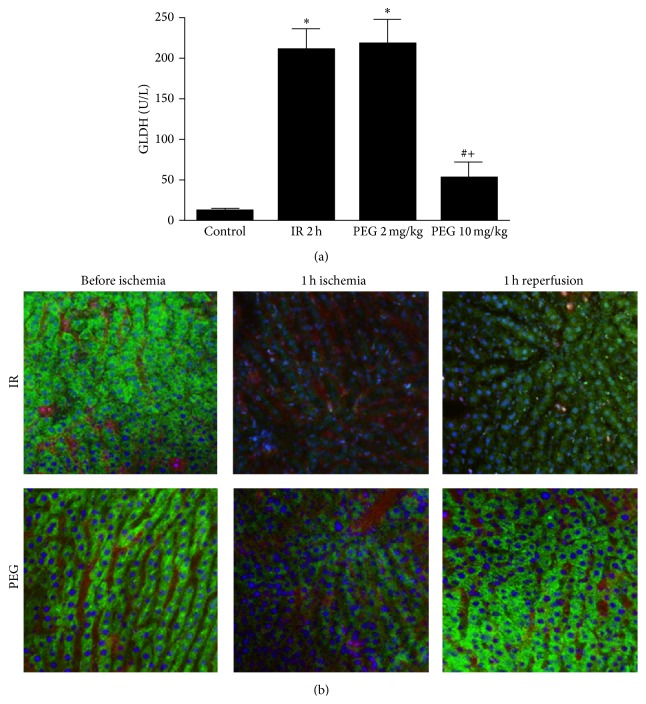
Mitochondrial damage after liver ischemia reperfusion. PEG 35 at 10 mg/kg decreases glutamate dehydrogenase (GLDH) levels (a) and preserves mitochondrial polarization status (b) (mitochondrial membrane potential dye Rhodamine 123 (green color), the nuclei dye Hoechst (blue color), and the plasma albumin dye Evans blue (red color)). Data represent mean ± SEM. ^*∗*^ *P* < 0.05 versus Control; ^#^ *P* < 0.05 versus IR 2 h.

It is well known that hepatic damage after reperfusion is associated with mitochondrial alterations. For this reason, we measured GLDH activity to assess mitochondrial injury after two hours of reperfusion. As indicated in Figure 2(a), the increase in GLDH levels observed in ischemic group was prevented in PEG 10 mg/kg group. No changes were observed in PEG 10 mg/kg versus Control. By contrast, pretreatment with PEG 35 at 2 mg/kg was not sufficient to protect mitochondria against IRI. Also, we performed intravital multiphoton microscopy in living rats at one hour of reperfusion to evaluate mitochondrial polarization status using Rh123 (Figure 2(b)). In the livers of Control rats, bright punctate Rh123 fluorescence was observed representing cells with polarized mitochondria. However, livers from ischemic rats showed a dimmer diffuse cytosolic fluorescence indicating mitochondrial depolarization. Importantly, when rats were pretreated with PEG 35 at 10 mg/kg, mitochondrial depolarization was lessened after ischemia and totally reverted after reperfusion.

**Figure 3:**
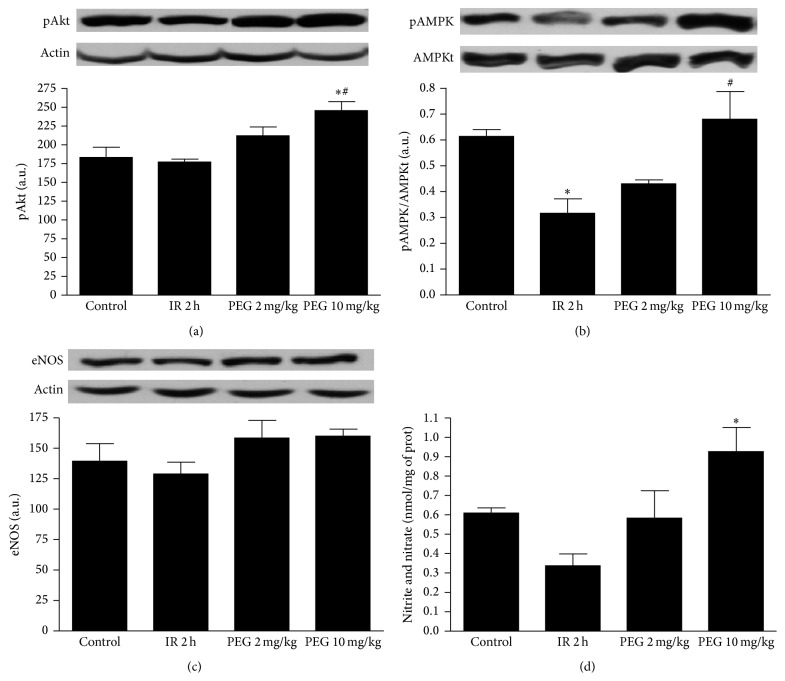
Effect of PEG 35 on Akt, AMPK, eNOS activation, and NO generation. PEG 35 preconditioning at 10 mg/kg enhances AMPK and Akt activation and increases nitrite/nitrate levels without any effect on eNOS. Western blot and densitometric analysis of phosphorylated Akt/b-actin (a), phosphorylated AMPK/total AMPK (b), eNOS/b-actin (c), and biochemical determination of nitrite+nitrate levels in liver tissue (d). Data represent mean ± SEM. ^*∗*^ *P* < 0.05 versus Control; ^#^ *P* < 0.05 versus IR 2 h.

Next, we investigated the potential signalling mechanisms involved in the beneficial effects of PEG 35 pretreatment. In this sense, it has been reported that protein kinase B (Akt) is a prosurvival protein that decreases apoptosis in models of IRI [13]. Also, it is well known that AMPK is a cellular metabolic sensor that switches the cell to an energy conserving status under ischemic conditions [14]. Our results show that PEG 35 administration at the dose of 10 mg/kg induced a significant activation of both Akt and AMPK (Figures 3(a) and 3(b), resp.).

Both AMPK and Akt have been shown to activate endothelial nitric oxide synthase (eNOS) [15]. However, no changes in eNOS activation were found when PEG was used (Figure 3(c)) although significant increases in nitrites/nitrates levels in liver tissue were observed (Figure 3(d)).

**Figure 4:**
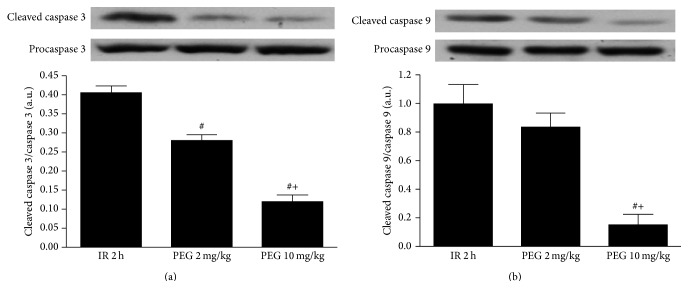
Effect of PEG 35 on liver apoptosis after ischemia reperfusion. PEG 35 at 10 mg/kg reduced the levels of apoptotic proteins caspases 3 and 9. Western blot and densitometric analysis of cleaved caspase 3/procaspase 3 (a) and cleaved caspase 9/procaspase 9 (b). Data represent mean ± SEM. ^#^ *P* < 0.05 versus IR 2 h; ^+^ *P* < 0.05 versus PEG 2 mg/kg.

Given the central role played by apoptosis in liver IRI, we evaluated the effects of PEG 35 on hepatic apoptosis by measuring caspases 3 and 9. As shown in Figure 3, PEG 10 mg/kg promoted a significant reduction of cleaved caspases 3 and 9. No significant differences were found regarding pretreatment with PEG 35 at 2 mg/kg although a tendency towards a decrease was observed when compared to IR group (Figure 4).

**Figure 5:**
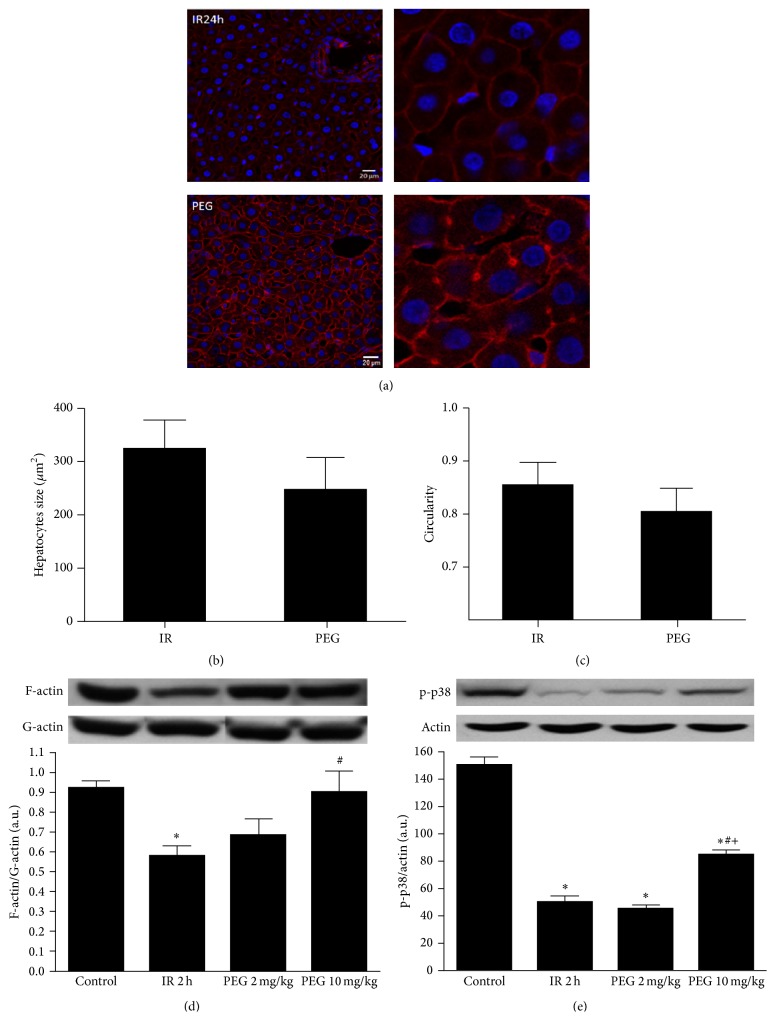
PEG 35 preconditioning contribution to cytoskeleton preservation. Images of confocal microscopy show that, in PEG 35 pretreated livers, filamentous actins (red) and hepatocytes morphology were better preserved when compared with nontreated ones. Also, PEG 35 pretreatment at 10 mg/kg enhances significantly F-actin/G-actin ratio and phospo-p38 protein levels. Confocal microscopy for F-actin (a) and determination of hepatocyte size (b) and hepatocyte circularity (c) and western blot and densitometric analysis of F-actin/G-actin (d) and phosphorylated p38 (e). Data represent mean ± SEM. ^*∗*^ *P* < 0.05 versus Control, ^#^ *P* < 0.05 versus IR 2 h, and ^+^ *P* < 0.05 versus PEG 2 mg/kg.

In addition, we studied the potential PEG effects on cytoskeleton. Confocal microscopy images of F-actin stained with Phalloidin showed that, in PEG 35 pretreated livers, filamentous actin associated with the membrane microfilamentous network and the pericanalicular band are kept preserved (Figure 5(a)). Also, morphology of hepatocytes in ischemic livers was compromised after ischemia reperfusion as shown in quantification of hepatocytes size and circularity (Figures 5(b) and 5(c)). Increase in size and circularity indicates that hepatocytes have swollen and lost their shape which was less evident in case of PEG treated livers. Moreover, IRI induced an important decrease of F-actin/G-actin ratio, which was significantly prevented when PEG 10 mg/kg was administered, as it is indicated in Figure 5(d). Also, it has been reported that activation of p38 leads to cytoskeletal changes by increasing the hepatocyte F-actin content after IRI [16]. For this reason, we investigated whether the cytoskeleton preservation observed with PEG pretreatment was correlated with changes in p38 phosphorylation status. Indeed, our results showed that PEG preconditioning increased p38 activation (Figure 5(e)).

Finally, we performed intravital microscopy using PEG-FITC in order to study its localization in liver tissue. We observed that PEG was still present in liver vascular bed after one hour of ischemia and one hour of reperfusion, which is showed in the additional movie file (see Additional File 1 in Supplementary Material available online at http://dx.doi.org/10.1155/2016/9096549).

## 4. Discussion

IRI is an important cause of liver damage occurring during surgical procedures including hepatic resection and liver transplantation and represents the main cause of graft dysfunction and primary nonfunction after transplantation [17]. PEGs are water soluble nontoxic polymers that are known to play an important role in the cytoprotection against ischemic damage. Recent studies have shown that PEG exerts anti-inflammatory, antiapoptotic, immunosuppressive, and membrane stabilization effects [4, 5, 8, 18–20]. From this perspective, it is reasonable to expect that PEG administration may be an effective therapeutic strategy against IRI. In this study, we demonstrate, for the first time, that PEG 35 preconditioning protects rat liver against warm IRI.

We have focused our study on PEG with molecular weight of 35 kDa because it has been previously demonstrated that PEG 35 was effective in preventing cold IRI in liver when it was added to organ preservation solutions [21–23]. Moreover, PEG 35 has been shown to protect renal cells against cold ischemia [24]. Also, we recently evidenced that PEG 35 addition to washout solution protected cold stored livers against reperfusion injury [25]. However, PEGs with different molecular weights such as PEG 8 [11] or PEG 20 [8, 9] might also be useful for conferring protection against IRI.

In order to achieve an efficient hepatoprotection, the most suitable concentration of PEG 35 was 10 mg/kg. This concentration was well tolerated as Control rats injected with PEG 35 at 10 mg/kg did not present any liver damage (data not shown).

The beneficial effects induced by PEG 35 are mainly associated with the preservation of the mitochondrial status, as revealed by decreases in GLDH levels and intravital microscopy findings. Since mitochondria are sensitive targets for damage during IRI [23, 24], the lessened hepatic injury observed when PEG 35 was administered at 10 mg/kg coincided with increased mitochondrial preservation.

We next determined whether PEG 35 beneficial effect could be related to the activation of protective cell signalling pathways. Our results showed that PEG 35 at 10 mg/kg contributes to AMPK and AKT activation. These facts are in line with previous reports showing that PEG 20 protects against heart ischemia through AKT activation [7, 8] and PEG 35 protects rat liver against reperfusion injury, in part, through AMPK activation [9]. These observations suggest that PEG protective effects are not only related to its known role as an oncotic support but also related to its pharmacological properties.

Akt and AMPK activation have been related to apoptosis inhibition in many models of IRI [15, 26, 27]. As it was expected, PEG 35 at 10 mg/kg prevented caspase 3 and caspase 9 activation. These observations are in line with results observed by Malhotra et al. who demonstrated that PEG 15–20 protected cardiac myocytes from hypoxia and reoxygenation induced apoptosis [8]. Moreover, we have previously reported that PEG 35 addition to preservation solutions acts as an oncotic agent ameliorating organ graft preservation by reducing apoptosis in rat liver transplantation [28].

Nitric oxide (NO) is a gaseous vasodilator implicated in the regulation of hepatic microcirculation, which is impaired upon IRI [25]. In this sense, we found that PEG 35 at 10 mg/kg significantly increased NOx levels and this was not correlated with eNOS activation. This observation is concomitant with a previous published report showing that PEG induced arteriolar dilatation which was not correlated to eNOS activation [26]. However, we have recently evidenced that the benefits of PEG in IRI were associated closely with eNOS activation [8, 9]. Thus, more investigations are needed to elucidate the precise mechanisms of NO generation mediated by PEG.

Structural alterations of the cytoskeleton following ischemia reperfusion have been reported to cause disturbances of intracellular transport processes and cell motility and microcirculation leading to organ dysfunction [29–32]. In liver cells, F-actin is a relevant component of liver cytoskeleton which forms microfilaments involved in intracellular transport processes, such as exocytosis and endocytosis, maintenance of cell shape, and canalicular motility responsible for bile flow [25, 31, 33, 34]. In this context, we have explored whether PEG 35 pretreatment could maintain the cytoskeleton structure and preserve the morphological characteristics of hepatocytes. Indeed, our present data confirmed that F-actin/G-actin ratio is increased as a consequence of PEG administration at 10 mg/kg. Furthermore, confocal microscopy findings confirmed that PEG contributes to the regulation of endothelial cell barrier by rearranging the actin cytoskeleton. Hepatocytes presented a more normal hexagonal morphology in livers pretreated with PEG 35 compared with livers submitted to IRI. All of these observations are consistent with a recent study in lung endothelial cells evidencing that PEG 15–20 preserves the architecture of the endothelial cytoskeleton [35]. Moreover, it has been demonstrated that PEG induced membrane stabilization through sarcolemmal lipid-raft architecture preservation [8]. These published data suggested that PEG interaction with cell membrane (adhesion or intercalation) preserved the cytoskeleton. In our study we further evidenced that PEG-induced-p38 MAPK activation may also be responsible for cytoskeleton preservation. However, the precise mechanisms of how PEG could affect cytoskeleton remain to be elucidated.

Currently, the proposed strategies against IRI rely on surgical procedures such as IPC or on the use of pharmacological agents (pharmacologic preconditioning) [36]. IPC is a well-established technique that consists of the application of brief episodes of ischemia and reperfusion which cause protection against the subsequent prolonged ischemic insult [37]. However, this manipulation is not tolerated in most operation rooms. Pharmacologic preconditioning consists of the administration of drugs that block injurious pathways directly or trigger endogenous protective mechanisms [36]. Although most of these drugs were effective in reducing IRI in many experimental models, studies that evaluate their efficacy in the clinical settings are still lacking. Moreover, their benefits are limited to the specific drug activity and their potential adverse effects. Compared to IPC and pharmacologic preconditioning, PEG presents the advantages of being safe and multitarget drug. Indeed, PEG effects are associated with the majority of the events occurring during IRI such as oxidative stress, mitochondrial preservation, cytoskeleton protection, and the induction of prosurvival and cytoprotective signaling pathways.

## 5. Conclusions

In conclusion, the present work evidences that intravenous administration of PEG 35 is a useful tool for liver preconditioning against the deleterious effects of IRI. Based on these findings, PEG 35 administration could be a useful approach in clinical settings.

## Supplementary Material

PEG conjugated with FITC (green color) is present in liver vascular bed after one hour of ischemia and one hour of reperfusion.
